# Supporting Careers of Women in Clinical Immunology: From Conceptualization to Implementation

**DOI:** 10.3389/fped.2022.864734

**Published:** 2022-03-29

**Authors:** Victoria R. Dimitriades, Alexandra F. Freeman, Sarah E. Henrickson, Roshini S. Abraham

**Affiliations:** ^1^Division of Pediatric Allergy, Immunology and Rheumatology, Department of Pediatrics, University of California Davis Health, Sacramento, Sacramento, CA, United States; ^2^Laboratory of Clinical Immunology and Microbiology, National Institute of Allergy and Infectious Diseases, National Institutes of Health, Bethesda, MD, United States; ^3^Division of Allergy and Immunology, Children's Hospital of Philadelphia, Department of Pediatrics, Perelman School of Medicine, University of Pennsylvania, Philadelphia, PA, United States; ^4^Department of Pathology and Laboratory Medicine, Nationwide Children's Hospital, Columbus, OH, United States

**Keywords:** women, immunology, mentor, leadership, diversity

## Introduction

Women have proven themselves integral to academic and clinical excellence, diversity, and all aspects of the missions of various immunology societies. Yet, women in Immunology still face challenges in academia and leadership positions, as many others have noted in medical and research specialties, with resultant attrition from the advancement pipeline (1). A concerted effort and thoughtful strategies are required to support the careers of women across biomedical science and medicine, to ensure the success of women in all aspects of academic, research, and clinical life (2). While many strategies warrant consideration in this process, the conscious effort to promote gender balance by academic medical and scientific journals and conference organizing committees can help to expand not only the high-quality presentations of valuable work, but may thereby facilitate promotion of women as members of editorial boards, professional committees, and in other invited positions (3, 4).

In early 2017, with the primary goal to support and encourage the promotion of women at all levels of training and career advancement, the Clinical Immunology Society (CIS) launched their Women in Clinical Immunology Sciences (WCIS) committee. This committee included members at various stages of their careers, with the intentional recruitment of all genders. The committee's purpose was to help current and future members to attain their full academic potential *via* mentorship, sponsorship, professional development, and leadership, in an inclusive and supportive climate.

## Surveying the Membership

For our first undertaking in understanding the composition and needs of the group, we conducted a survey of the entire CIS membership with the goal of tailoring programming to the needs of current members (Supplementary Material 1). Out of a total membership cohort of 717, we had 155 respondents (22%), with 72% identifying as female and 28% identifying as male (no respondents identified as another gender). These proportions were different compared to the general membership, which identified as 50% female and 50% male at that time, likely reflecting the interest in the target group being addressed. Of the members who took part in the survey, the female respondents tended to be younger (73% <45 yr, 8% >55 yr) while the male respondents tended to be older (42% <45 yr, 23% >55 yr). This was also reflected in their career levels, with the female respondents predominantly at the trainee and assistant professor levels (61%), while the male respondents were predominantly at the associate and full professor levels (58%).

It was relevant to note that issues perceived as barriers to *women in the sciences* was similar in both groups of respondents, centering around balancing work and life goals, gender biases, concerns for childcare and support mechanisms, and ability to get grant funding. This finding was reflective of general concerns, which have been articulated for female faculty in medical schools as well (5).

Both gender groups reported low familiarity of their institution's policies on career flexibility practices (if any), though both genders reported taking leave for childcare (variable by gender- most females <12 weeks, most males <2 weeks). Of particular note, in these groups, the male respondents reported utilizing a larger proportion of elder care leave (14% male vs. 6% female), reflecting a growing discussion around support for faculty with expanded caregiving responsibilities (6).

Finally, as has been noted in other fields as well, there was a paucity of female senior mentors or role models in both groups, with 40% of females and 30% of males reporting no access to female role models in their specialty.

## Setting Plans in Motion

With the results of this membership survey in hand, and taking cues from other scientific societies with similar goals, we set up a plan to advocate for the four identified strategic priority areas, and to support further scholarship to address pipeline issues and barriers on the path of leadership and success for women in Clinical Immunology.

### Awareness and Education

It has been demonstrated previously that while women start as 50% of the population in the clinical sciences, those numbers decrease notably as the years pass (7). Indeed, at the time of our survey, we were very pleased to note that our strong representation in the general membership of our society was in line with this overall number. However, a more granular review of our survey respondents showed the same decrease in numbers which was apparent across the clinical sciences over time: only 13% of the female respondents reported being in the Clinical Immunology field for more than 20 years compared to 40% of the male respondents. It was clear that a level of investment was needed to enhance the experience and opportunities for women earlier in their chosen path. With the first goal of increasing retention of women within our field, we set out to create a visible entity which would support women from their initial foray into the field of clinical immunology though the remainder of their careers. The official capacity of our group, which was assembled with members who expressed interest in this same goal, created an awareness for other interested individuals and committees to connect with us for ideas and resources.

We developed a web-based hub with the ability to disseminate information and serve as an on-line source for events and resources. Since networking was one of the most effective ways to bring awareness to our goals, we set up sessions at our Annual meetings, which included panels and educational discussions as well as time to interact with other members in order to make personal connections around the topics. We made a concerted effort to invite all members to attend and participate in these events, as establishing male Allyship has been a proven catalyst for change as well (8). Even during times of altered in-person attendance, curated networking meetings allowed us to discuss pertinent issues, both in a large-group and small-group setting. Establishing our group as a support mechanism for trainees will also be a useful tool for engagement and retention in the future.

### Mentorship and Sponsorship

Mentorship in academic medicine has been reported to increase personal and career development, increase job satisfaction, and increase research productivity, including publications and grant awards (9). With this in mind, we facilitated the involvement of female mentors though the CIS Mentoring program, which pairs senior and junior members and permits the former to offer guidance and support to the latter. However, it must be noted that dyad mentoring relationships are not necessarily the most successful model for women, who also thrive with multiple mentor relationships or peer-mentor groups (10). To this end, we are preparing a support mechanism for those currently working on scholarly projects and grant preparations where they can get advice from others going through (or who have recently been through) the same process. Finally, as we recognize that true mentorship comes in many forms, it is important to acknowledge that the best mentoring for women does not always come from women. Insightful and purposeful mentoring, within in the context of differential gender impacts, can and should be delivered by mentors of any gender.

While mentorship is essential for career development, advancement in one's career can truly be facilitated by *sponsorship*, which embodies a different concept from mentorship. Distinct from the advisory role of a mentor, sponsorship requires using influence to provide high-profile opportunities which can lead to career success (11). This has been shown to be influenced by gender, with women in academics noted to have fewer sponsorship opportunities and experiences (12). To this end, WCIS, in partnership with our Early Career Immunologists (ECI) group, launched an updated database feature to help facilitate sponsorship of all members, and to highlight them for consideration of roles on Scientific Boards, as Chairs of committees, for leadership and editorial positions, and as speakers for conferences and company events. We hope to continue to develop this feature so that it can be used as a resource for those outside our group in order to identify ideal members for their needs. The support of this initiative has lasting benefits, which includes not only career advancement for everyone involved, but also the added benefit of promoting retention of women in academic specialties through career support.

### Culture and Resilience

Understanding the underlying issues which impact women in Clinical Immunology can help to design strategies to address the barriers to equal and diverse representation in our field. Moreover, specific policy implementations which focus on career sustainability, academic collaboration, compensation/funding, and valuation of work can signal a commitment to equity planning within a professional organization (13). In order to better inform ourselves of the current climate as it relates to our work and research, our Annual Meeting programming early in the conception of WCIS included an introspective review of the topic of Implicit Bias and Diversity in Academic medicine. This session was a great success as it brought together all meeting attendees to begin the conversation on this important work. We also assessed the current state of our field through programming which reviewed gender and diversity in the field of Clinical Immunology (and the related field of Allergy). Through review of the literature as well as interactive sessions with authors of recent articles addressing this issue, we were able to identify specific goals for future impact (14).

Active participation of women in committees for scientific programming or hiring has been shown to increase the diversity and functioning of the group (15). To highlight the importance of this aspect in our field, we promoted programming that focused on the empowerment of women through local governance involvement and access, which ultimately can help to influence shifts in culture and policy advancement. Through real world examples of how women are increasing their representation in voting and in local government positions, the measurable impacts on congressional initiatives and goals are empowering for those looking to make a difference.

During the last 2 years, especially in the context of the pandemic, the need for resilience programming has increased greatly. Lack of career flexibility, the burden of family care, and the feeling of burnout have been issues which have been known to cause reduction in professional work effort at many levels (16). When asked, two-thirds of our female survey respondents felt that they were not achieving the balance they needed in their career and home lives. This issue has been exacerbated by the current global crisis, though it is germane to note that the perception of support and tangible demonstrations of appreciation by the individual's institution or organization has a more positive effect on career planning (17). To further explore this area, future programming will include identification of resources and networks of support to help members manage issues of self-care and career balance.

### Leadership/Faculty Development

Women who are exposed to highly successful figures displayed more empowered behavior, and were critically perceived as being more empowered, with more positive self-reflective experiences (18). By encouraging strong female role models, we can increase their visibility and empower other women on their path to leadership. With this in mind, we established the “Women Pioneers of Immunology” feature on our Society website to highlight women who had taken strong leadership roles in our field and served as role models for others. Each individual was able to tell her story to bring awareness to the myriad of pathways which one can take to career and personal success.

However, our survey also indicated that many women felt that they were not adequately prepared to take on some of these leadership roles. In fact, leadership training programs targeting female faculty have reported positive benefits such as skills building, increased representation, promotions, retention, and remuneration, which can all play a role in promoting gender equity (19). With this in mind, we developed virtual programming through partnerships with several successful leaders in our society who helped us to develop our leadership skills though education sessions reviewing *Time management, Organizing and Leading a Team*, and *Negotiations*.

## The Path Forward

We recognize that progress is often incremental and the initiatives outlined above represent the first steps in acknowledging gender inequity issues and the need to uplift all members of our Clinical Immunology community. In particular, we would like to make sure that programs are put into place which utilize varying avenues of support for the women in Clinical Immunology who continue to be under-represented as they progress into their careers. Furthermore, it has been a natural progression for the WCIS to partner with the Diversity & Inclusion Committee of CIS to develop and offer new programming, which will expand the presence of both inherent and acquired diversity in our membership (20). While the intrinsic nature of supporting women lends itself to a binary characterization, we must also keep awareness and encourage further research into the impact of gender disparities in the sciences on non-gender or multi-gender identifying members of our groups. Finally, we need to keep in mind that inclusion requires both affirmation and action, which should be approached simultaneously at many levels. Thus, we hope that by sharing our experience in the field of Clinical Immunology through the perspective of the Clinical Immunology Society (CIS), we can embolden others in related professional organizations to initiate or enhance their current programming, in order to make Clinical Immunology a welcoming and nurturing discipline, accommodating a plethora of ideas and opportunities for all of our members.

## Data Availability Statement

The raw data supporting the conclusions of this article will be made available by the authors, without undue reservation.

## Author Contributions

VD conceptualized and interpreted the survey and wrote the manuscript. AF and SH conceptualized and interpreted the survey and edited the manuscript. RA conceptualized the survey and edited the manuscript. All authors contributed to the article and approved the submitted version.

## Conflict of Interest

The authors declare that the research was conducted in the absence of any commercial or financial relationships that could be construed as a potential conflict of interest.

## Publisher's Note

All claims expressed in this article are solely those of the authors and do not necessarily represent those of their affiliated organizations, or those of the publisher, the editors and the reviewers. Any product that may be evaluated in this article, or claim that may be made by its manufacturer, is not guaranteed or endorsed by the publisher.
