# Supplementary material for: Supporting Careers of Women in Clinical Immunology: From Conceptualization to Implementation
Source: Front Pediatr. 2022 Mar 29;10:864734. doi: 10.3389/fped.2022.864734 (PMC9002117; doi:10.3389/fped.2022.864734)
Supplement: Supplementary file 1 [file Data_Sheet_1.pdf]

Supplementary Material S1: WCIS survey questions

1. Please indicate your gender
    a. Male
    b. Female
    c. Other (or Do not identify, Prefer not to say)
2. Which best represents your racial or ethnic heritage:
    a. African American
    b. East Asian
    c. Caucasian
    d. South Asian
    e. Pacific Islander
    f. Middle Eastern
    g. Hispanic/Latino
    h. Native American
    i. Other
3. In what part of the world are you located?
    a. North America
    b. Central America
    c. South America
    d. Europe
    e. Africa
    f. Middle East
    g. Asia
4. Please indicate the degree which you have or are working towards:
    a. MS
    b. MD
    c. PhD
    d. MD/PhD
    e. RN
    f. None
    g. Other
5. In what capacity are you employed?
    a. University
    b. Government
    c. Non-profit Research Center
    d. Private practice
    e. Laboratory
    f. Industry
    g. Other
6. If at University, please note your position
    a. Instructor
    b. Assistant Professor

    c. Associate Professor
    d. Professor
    e. Other

7. If in Private Practice, are you
    a. Junior partner
    b. Senior partner
    c. Employee of practice
    d. Other
8. Do you work full time or part-time?
    a. Full time
    b. Part time (at any percentage)
9. How much of your time is spent doing direct patient care?
    a. <25%
    b. 25-50%
    c. 50-75%
    d. >75%
10. Are you married or in a long-term relationship?
    a. Yes
    b. No
11. Which best describes your spouse's or partner's work?
    a. Works in the sciences
    b. Works in a non-science field
    c. Stay at home spouse/partner
    d. Currently between jobs
    e. Do not have spouse/partner
    f. Other
12. Do you have children?
    a. No
    b. Yes, under 18 years of age
    c. Yes, older than 18 years of age
    d. Yes, have children older and younger than 18 years of age
13. Did you take leave for the birth of your child(ren)? If so, how many weeks?
    a. Maternity
        i. Child #1 ____
        ii. Child #2 ____
        iii. Child #3 ____
    b. Paternity
        i. Child #1 ____
        ii. Child #2 ____
        iii. Child #3 ____
14. Have you ever taken leave for elder care?
    a. Yes
    b. No
15. How many years have you been in the field of immunology, since your training?

    a. <5 years
    b. 5-10 years
    c. 10-15 years
    d. 15-20 years
    e. >20 years

16. Do you currently serve on any boards or committees of local or national science committees?
    a. Yes
    b. No
17. Which of the following issues do you feel are barriers currently facing women in the sciences?
    a. Grants/funding
    b. Laboratory space
    c. Child care support
    d. Gender biases
    e. Access to mentors
    f. Lack of role models
    g. Balancing Life goals
    h. Having/raising children
    i. Elder care
    j. Low pay
    k. Scarcity of Job openings
    l. Other (specify)
18. Regarding the above barriers, which are the three most significant ones?
    a.
    b.
    c.
19. Does your workplace have a policy on career flexibility?
    a. Yes
    b. No
    c. Not sure
20. How well to you feel that you manage your career/work and home/family obligations?
    a. Great, I have harmony in all places
    b. Pretty good, I am balanced in my goals and outcomes for several aspects of my life
    c. Just OK, I always have loose ends which never get tied up
    d. Awful, I have guilt for not being able to spend more time meeting all of my obligations
21. What percentage of your department or work/peer group are females?
    a. <10%
    b. 10-25%
    c. 25-50-%
    d. 50-75%
    e. >75%
22. Do you have female senior mentors or role models?
    a. No
    b. Yes, less than 5 total
    c. Yes, more than 5 total

23. Have you personally experienced gender bias at work, even if not blatently?
    a. Yes
    b. No
    c. Not sure
24. Have you passed up or been excluded from a career opportunity or advancement due to any of the barriers mentioned in question #14?
    a. Yes
    b. No
    c. Not sure
25. Would you be interested in programming through the CIS regarding:
    a. Life Balance, Mindfulness and Resilience in the Sciences
        i. Yes
        ii. No
    b. Pathways to Leadership
        i. Yes
        ii. No
    c. Finding and building a good Mentor/Mentee relationship
        i. Yes
        ii. No
